# The Bed Nucleus of the Stria Terminalis as a Brain Correlate of Psychological Inflexibility in Fibromyalgia

**DOI:** 10.3390/jcm9020374

**Published:** 2020-01-30

**Authors:** Albert Feliu-Soler, Ignacio Martínez-Zalacaín, Adrián Pérez-Aranda, Xavier Borràs, Laura Andrés-Rodríguez, Juan P. Sanabria-Mazo, Nicolás Fayed, Christian Stephan-Otto, Christian Núñez, Carles Soriano-Mas, Juan V. Luciano

**Affiliations:** 1 Institut de Recerca Sant Joan de Déu, 08950 Esplugues de Llobregat, Spain; a.feliu@pssjd.org (A.F.-S.); l.andres@pssjd.org (L.A.-R.); csephanotto@pssjd.org (C.S.-O.); c.nunez@pssjd.org (C.N.); 2 Teaching, Research & Innovation Unit, Parc Sanitari Sant Joan de Déu, 08830 St. Boi de Llobregat, Spain; 3 Faculty of Psychology, Universitat Autònoma de Barcelona, 08193 Bellaterra (Cerdanyola del Vallès), Barcelona, Spain; xavier.borras@uab.cat (X.B.); juanpablo.sanabria@e-campus.uab.cat (J.P.S.-M.); 4 Department of Psychiatry, Bellvitge University Hospital, Bellvitge Biomedical Research Institute-IDIBELL, 08907 Barcelona, Spain; imartinezz@outlook.es (I.M.-Z.); csoriano@idibell.cat (C.S.-M.); 5 Department of Clinical Sciences, School of Medicine, University of Barcelona, 08036 Barcelona, Spain; 6 Department of Radiology, Quirónsalud Hospital, 50012 Zaragoza, Spain; nicola33fr@hyahoo.es; 7 Centro de Investigación Biomédica en Red de Salud Mental (CIBERSAM), 28029 Madrid, Spain; 8 Department of Psychobiology and Methodology of Health Sciences, Universitat Autònoma de Barcelona, 08193 Barcelona, Spain

**Keywords:** fibromyalgia, psychological inflexibility, mindfulness, bed nucleus of the stria terminalis, neuroimaging

## Abstract

This study explored the brain structural correlates of psychological flexibility (PF) as measured with the Psychological Inflexibility in Pain Scale (PIPS) in patients with fibromyalgia (FM). Structural magnetic resonance imaging data from 47 FM patients were used to identify Gray Matter Volume (GMV) alterations related to PIPS scores. Brain GMV clusters related to PIPS were then correlated with clinical and cognitive variables to further explore how emerged brain clusters were intertwined with FM symptomatology. Longitudinal changes in PIPS-related brain clusters values were assessed by studying pre–post data from 30 patients (15 allocated to a mindfulness-based stress reduction (MBSR) program and 15 to treatment-as-usual). Changes in PIPS-related brain clusters were also explored in participants showing greater/lower longitudinal changes in PIPS scores. PIPS scores were positively associated with GMV in a bilateral cluster in the ventral part of the bed nucleus of the stria terminalis (BNST). Significant associations between BNST cluster with functional impairment, depressive symptomatology, perceived stress and the nonjudging mindfulness facet were observed. Participants reporting greater pre–post increases in PIPS scores showed greater increases in BNST cluster values. These findings contribute to the understanding on the neurobiological bases of PF in FM and encourage further explorations of the role of the BNST in chronic pain.

## 1. Introduction

The neural underpinnings of third-wave cognitive behavioral therapies, such as mindfulness and acceptance-based interventions, have become a topic of burgeoning interest in recent years [1,2,3,4]. Many studies have reported significant relationships between mindfulness and brain structures involved in attention regulation, body awareness, emotion regulation, and perspective taking. Such associations have been found when studying mindfulness as a trait [5,6,7,8,9,10,11] or as a practice, in studies comparing meditators and non-meditators or in studies looking for brain changes following mindfulness-based interventions [12,13]. Associations between mindfulness, brain structure (i.e. gray matter, white matter), and function have been found in multiple brain regions, including the anterior and posterior cingulate cortex/precuneus, insula, temporo–parietal junction, different areas in the prefrontal cortex, hippocampus, and the amygdala, suggesting an involvement of a large-scale neural network conveying the effects of mindfulness [3,4,12,13,14].

By contrast, other psychological constructs also likely to underpin the effects of third-wave cognitive behavioral interventions on health have received considerably less attention. Psychological (in)flexibility (PF) is a key construct in Acceptance and Commitment Therapy (ACT) [15] and mindfulness-based interventions and provides a framework for understanding the impact of chronic pain on an individual’s functioning [16]. PF is defined as the ability to act effectively and in accordance with personal values even in the presence of difficult experiences such as negative thoughts and emotions or unpleasant body sensations (e.g. pain). This construct comprises six core processes, including mindfulness, acceptance, decentering, self as a context, accordance with personal values and committed action [15,17,18].

PF appears to be an important contributor in multiple mental health and medical conditions [19], including chronic pain [20]. Many studies evaluating PF components have consistently found that these are highly related to functioning and quality of life in patients with chronic pain, with higher flexibility being related to better outcomes [16,21,22]. Similarly, higher PF has also been found to be associated with less pain intensity and interference, less anxiety and depressive symptomatology, and improved physical and mental functioning in patients with FM [17,23,24]. Furthermore, PF (and its individual components) have also been reported to mediate clinical changes in acceptance- and mindfulness-based interventions delivered to patients with chronic pain [22,25] and FM [26,27,28].

Although PF has been established as a relevant transdiagnostic mechanism and as a key variable in explaining the effects of third-wave cognitive behavioral interventions on health, studies assessing the brain structures underpinning this set of cognitive processes are lacking [25]. The main objective of the present study was to explore the brain structural correlates of PF, as measured by the Psychological Inflexibility in Pain Scale (PIPS) [24] in patients with FM. We used voxel-based morphometry, a well validated technique that facilitates exploration of volume changes throughout the brain, including cortical and subcortical areas [29]. Here we focus on Gray Matter Volume (GMV), which is the most frequently evaluated brain structural correlate of mindfulness in cross-sectional designs (e.g., [10]). We also examined how brain volume clusters emerging from this analysis related to other clinically relevant and third-wave cognitive variables (i.e., mindfulness and self-compassion) in FM. Finally, we also examined the effects of an intervention known to promote PF in patients with FM, namely mindfulness-based stress reduction (MBSR) [27], compared to treatment-as-usual (TAU) on PIPS-related brain volumes, in a longitudinal study performed in a subset of participants. The clinical impact of the present research is two-fold: To broaden our knowledge on the biological correlates of cognitive processes (i.e., PF) relevant for chronic pain and offer potential new brain-based biomarkers underpinning PF that could in future be targeted with psychological interventions or be useful as prognostic/predictive factors in the field of precision medicine.

## 2. Materials and Methods

### 2.1. Study Sample

The sample comprised 47 women [Mean age: 51.6 (SD= 7.6; range 35–64 years)] with a FM diagnosis according to the American College of Rheumatology (ACR) 1990 criteria recruited at the Rheumatology service of Parc Sanitari Sant Joan de Déu (St. Boi de Llobregat, Spain). All participants were part of the EUDAIMON project, a randomized controlled trial (RCT) on the efficacy, cost-utility and biological underpinnings of MBSR for patients with FM [27,30]. All patients were female and right-handed, and the following exclusion criteria were used: Presenting acute pain unrelated to FM on the day of the scan, having changes in medication in the last three days, presenting a body mass index (BMI) > 36 Kg/m^2^ or weight > 110 Kg or other standard magnetic resonance imaging (MRI) exclusion criteria (e.g., pace-makers, metal implants), or being in litigation with the hospital.

### 2.2. Procedure

**Figure 1:**
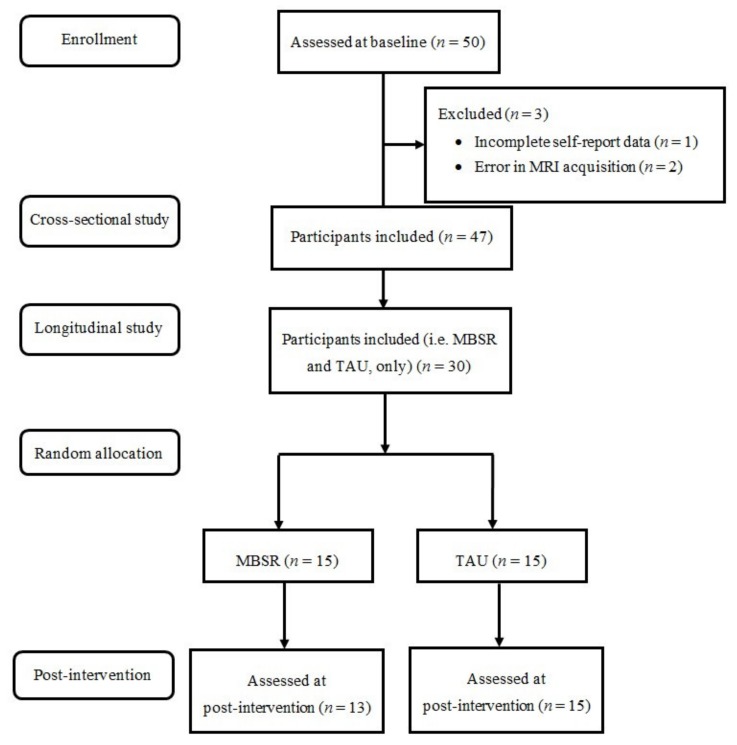
Flowchart of the study. MBSR: Mindfulness-based stress reduction; TAU: Treatment-as-usual.

Structural MRI data from 47 FM patients, prior to any treatment intervention were used to identify GMV alterations related to PIPS scores. Additional post-treatment GMV data from 30 of the patients (15 followed the MBSR program and 15 followed TAU were also analyzed) see Figure 1 for the flowchart of the study. All patients signed an informed consent and participated voluntarily in the present study without an economic compensation. The Ethics Committee at the Sant Joan de Déu Foundation approved the study (PIC-102-15), which was performed in accordance with the ethical standards (1964 Declaration of Helsinki and following updates). The detailed protocol of the EUDAIMON study can be found elsewhere [30].

### 2.3. Measures

#### 2.3.1. Socio-Demographic Characteristics and Clinical History

The following information was collected: Gender, age, marital status, living arrangement, educational level, and employment status. Relevant clinical variables such as years since FM diagnosis and main comorbid psychiatric conditions were also evaluated.

#### 2.3.2. MRI Assessments

All scans were performed on a 3.0 T Phillips Ingenia MR scanner equipped with a 32-channel, phased-array receive-only head coil (Koninklijke Philips N.V., Amsterdam, Netherlands). At baseline, a high-resolution T1-weighted 3-dimensional volume scan was obtained for each participant with the following parameters: 233 slices; repetition time = 10.4 ms; echo time = 4.8 ms; flip angle = 8°; field of view = 24 cm; acquisition matrix = 320 × 320; voxel size = 0.75 × 0.75 × 0.75 mm. In the subgroup of participants with follow-up assessments, a second scan with identical parameters was acquired after ten weeks.

#### 2.3.3. Main Outcome Measure

The Psychological Inflexibility in Pain Scale (PIPS) [24] is a 12-item measure in a 7-point Likert-type scale (ranged from 1 = “never true” to 7 = “always true”), with higher scores indicating more psychological inflexibility to pain. The scale considers two PF facets applied to chronic pain: Pain avoidance (i.e., behavioral tendency to withdraw from planned and valued activities because of pain or its expectation; e.g., “I do not do things that are important to me to avoid feeling my pain”) and cognitive fusion to pain (i.e., identification with pain-related thoughts or difficulty in distancing oneself from these thoughts; e.g., “it is important to understand what causes my pain”). The internal consistency of the Spanish version of the PIPS was found to be good-to-excellent with Cronbach’s α for the total scale of 0.90 [17]. Only total PIPS scores were used in the present work.

#### 2.3.4. Clinical Measures

The Revised Fibromyalgia Impact Questionnaire (FIQR) [31] assesses functional impairment of the syndrome during the current week, with 21 items in a 11-point numeric rating scale from 0 to 10 (the higher score, the greater impairment) asking on three domains: physical function, overall impact and severity of symptoms. A FIQR total score (from 0 to 100) is obtained by adding the three domain scores together, with higher scores being indicative of a worse functional status. The Spanish version has demonstrated excellent reliability (α = 0.91 –0.95), adequate test-retest reliability of (*r* = 0.82), and good construct validity [32].

The Hospital Anxiety and Depression Scale (HADS) [33] includes 14 items (on a 0-to-3 scale each) evaluating the severity of anxiety (HADS-A) and depression (HADS-D) symptoms during previous week, with higher scores indicating greater severity. The HADS has shown good reliability (α = 0.80–0.85) and validity in Spanish patients with FM [20].

The Perceived Stress Scale (PSS) [34] is a 10-item (on a Likert-scale, from 0 = “never” to 4 = “very often”), self-administered scale which measures the degree to which respondents appraised daily life as stressful in the last month. The total score can range from 0 to 40. This scale is used worldwide for measuring perceived stress due to its adequate psychometric properties [35]. The Spanish version showed adequate internal consistency (α = 0.81), test–retest reliability (*r* = 0.73), validity, and sensitivity to change.

The Pain Catastrophizing Scale (PCS) [36] is a self-report measure composed of 13 items asking for the frequency of thoughts on a 5-point Likert scale (between 0 = “never” and 4 = “almost always”) about perceived catastrophic consequences of pain. It comprises three dimensions: Rumination (tendency to focus excessively on pain sensations), magnification (tendency to magnify the threat value of pain sensations), and helplessness (tendency to perceive oneself as unable to control the intensity of pain). Total scores may oscillate from 0 to 52. The Spanish version captures the three PCS dimensions with good reliability (α = 0.79), high test-retest reliability (*r* = 0.84), and sensitivity to change [37].

#### 2.3.5. Third-Wave Cognitive Measures

The Five Facet Mindfulness Questionnaire (FFMQ) [38] consists of 39 items assessing mindfulness in daily life in five different facets or aspects: (1) Observing or noticing internal and external experiences such as sensations, thoughts, and emotions; (2) describing or labelling internal experiences with words; (3) acting with awareness or focusing on one’s activities in the here and now; (4) nonjudging of inner experiences or taking a nonevaluative stance toward thoughts and feelings; and finally (5) nonreacting to inner experiences or allowing thoughts and feelings to come and go, without getting caught up or carried away by them. Items are rated on a Likert scale ranging from 1 (“never or very rarely true”) to 5 (“very often or always true”) and are distributed among five facets: Observing, describing, acting with awareness, non-judging, and non-reacting to inner experiences. All subscale scores range from 8 to 40, except non-reacting facet which ranges from 7 to 35 (higher scores indicating in each case a higher presence of corresponding mindfulness facet). The FFMQ facets have shown good reliability (with ω ranging from 0.82 to 0.92) and validity in Spanish subjects [39].

The Self-Compassion Scale-short form (SCS) [40] is a 12-item scale (in a Likert scale from 1 = “almost never” to 5 = “almost always”) designed to assess self-compassion across three dimensions: Common humanity (or seeing one’s suffering as part of the human condition, rather than as isolating), mindfulness (or being able to hold difficult feelings mindfully, rather than being over-identified with them), and self-kindness (or being kind rather than judgmental toward oneself). A total SCS score can be obtained by averaging each dimension score, with scores ranging from 1 to 5 with higher values indicating greater self-compassion. Despite its recent development, the SCS has shown adequate psychometric properties in Spanish samples with good reliability (α = 0.86) and high convergence with the original form (26-item version) of the SCS (*r* = 0.97) [41].

### 2.4. Data Analyses

For this study, only GMV was analyzed. Imaging data were pre-processed and analyzed with MATLAB version R2017b (The MathWorks Inc., Natick, MA, USA) and Statistical Parametric Mapping software (SPM12 [42]). Both for baseline and follow-up scans, we followed a standard voxel-based-morphometry-DARTEL (VBM-DARTEL; [43]) pipeline, consisting of tissue segmentation (gray and white matter and cerebrospinal fluid), generation of a DARTEL template for normalization to Montreal Neurological Institute (MNI) space, modulation for volume restoration and smoothing with an 8 mm full width at half maximum (FWHM) Gaussian kernel. Relationships between PIPS scores and voxel-wise GMV were assessed with a multiple regression model, with age and total GMV as nuisance covariates. Statistical significance was determined by a combination of voxel-level and cluster-extent thresholds, using the AlphaSim algorithm as implemented in the SPM REST toolbox (http://www.restfmri.net/forum/REST_V1.8). Input parameters to AlphaSim included a voxel-level probability of *p* < 0.001, a rmm of 5, the FWHM corresponding to the actual smoothing of the data after model estimation, and a mask volume consisting on a whole-brain mask of 832,388 voxels. We performed 1000 Monte Carlo simulations. The minimum spatial cluster extent (KE) to satisfy a family-wise error (FWE) rate correction of pFWE < 0.05 was 122 voxels. Moreover, the first eigenvariate of significant clusters (summarizing volume values across all voxels inside the cluster) was extracted for further exploration in SPSS 24.0 [44].

Associations between PIPS-related brain volumes with functional impairment, anxiety, depression, perceived stress, pain catastrophism, mindfulness facets, and self-compassion were computed by means of partial correlations (with age and total GMV as covariates). Bonferroni correction for multiple comparisons was applied (significance level set at *p* < 0.004) to better establish a gradation on the relevance of results. 

To evaluate treatment-induced volume changes in PIPS-related brain clusters after the MBSR program, a repeated-measures ANOVA comparing pre–post assessments (time) in MBSR vs. TAU (group) was conducted (age and change in total GMV were entered as covariates in the model). In order to also explore the extent to what changes in PIPS were related to changes in emerged brain clusters, delta scores (Δ) for PIPS scores were calculated (i.e., post minus pre scores) and then dichotomized into a new variable by using a median-split. A repeated-measures ANOVA was then conducted by using this dichotomous variable as independent variable and PIPS-related brain volumes (pre and post) as dependent variable (with age and change in total GMV as nuisance variables).

Except otherwise indicated (i.e., imaging analyses), all statistical analyses were computed using SPSS 24.0.

## 3. Results

### 3.1. Sample Descriptive Statistics

Descriptive statistics on clinical, third-wave measures and global GMV characteristics of the sample are displayed in Table 1.

### 3.2. Psychological Inflexibility Clusters in Voxel-Based Morphometry Analyses

**Figure 2:**
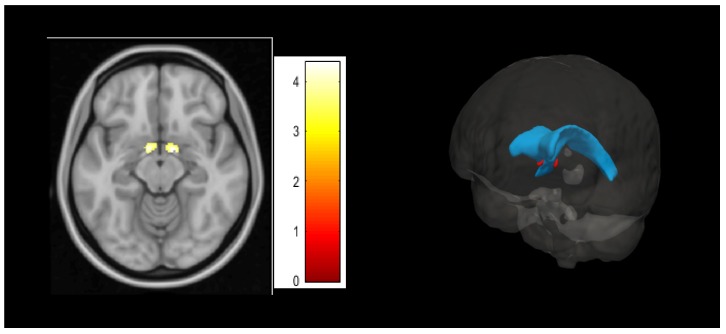
Left hand: Psychological Inflexibility in Pain Scale (PIPS)-related brain cluster (Montreal Neurological Institute (MNI) coordinates: x = 11, y= –2, z = –12 (right) and x = –8, y = 0, z = –11 (left)) in the bed nucleus of the stria terminalis (BNST). Right hand: Anatomical illustration of the BNST (red) and the lateral ventricles (blue).

Multiple regression analysis revealed that PIPS scores were significantly and positively associated with GMV in two bilateral brain clusters that corresponded to the ventral part of the bed nucleus of the stria terminalis (BNST) (peak MNI coordinates and KE: x = 11, y = –2, z = –12; T = 4.3748; 131 voxels (right) and x = –8, y = 0, z = –11; T = 4.3687; 164 voxels (left)) (Figure 2). To ascertain these clusters were located in the BNST, we used the Mai brain atlas [45], confirming that our results were indeed encompassing the ventral part (i.e., ventral to the anterior commissure) of the BNST. Although this part of the BNST is not included in some of the available BNST MRI masks [46], it is included in others [47].

### 3.3. Association between BNST Cluster with Clinical and Third-Wave Cognitive Measures

Significant positive associations (*p* < 0.05) with small-to-moderate effect sizes (*r* values ranging from 0.43 to 0.54) between ventral BNST cluster with all clinical variables (except HADS-A; *r* = 0.24, *p* = 0.106) were found at baseline. Regarding correlations with process measures, a positive significant association between BNST cluster and Observing facet was found (*r* = 0.35, *p* = 0.019), and significant negative correlations were found with Nonjudging (*r* = –49, *p* = 0.001), Nonreacting (*r* = –0.33, *p* = 0.028), and Act with Awareness (*r* = –0.30, *p* = 0.047) facets. A tendency to statistical significance was also found with Describing subscale (*r* = –0.26, *p* = 0.089). A significant association between BNST and self-compassion scores was also found (*r* = –0.34, *p* < 0.026). After applying Bonferroni’s correction for multiple comparisons (significance set at *p* < 0.004), the BNST cluster was still significantly correlated with FIQR, HADS-D, PSS, PCS, and Nonjudging mindfulness facet. For more details, see Table 2.

### 3.4. Changes in PIPS-Related Brain Volumes Across Time

Regarding volumes changes in the ventral BNST cluster, no significant group × time effects were found (*p* > 0.05). No effect of MBSR was found for PIPS scores either. See Table 3 for more details. 

**Figure 3:**
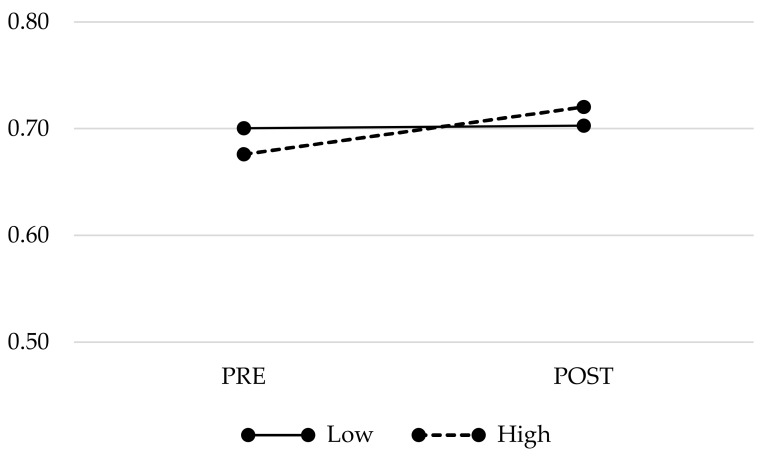
Pre-post gray matter volume’s changes in the BNST in patients presenting higher vs lower increases in Psychological Inflexibility Scale scores (i.e. “High” vs. “Low”).

Participants reporting higher increases in PIPS scores (i.e. “PF non-responders”; *n* = 15; mean (SD) ΔPIPS: 5.73 (8.81)) presented statistically significant increases in ventral BNST volumes (means (SD): PRE 0.68 (0.05), POST 0.72 (0.06)) compared with those with lower increases or even presenting decreases (i.e., “PF responders”; *n* = 13; mean (SD) ΔPIPS: –12.69 (7.93)) which they did present a very flat trajectory (means (SD): PRE 0.70 (0.05), POST 0.70 (0.07)) (group × time effect: F = 15.250; *p* = 0.001). For a graphical representation of the BNST pre–post trajectories, see Figure 3.

## 4. Discussion

The present study explored the structural brain correlates of psychological inflexibility in pain in a sample of patients with FM. A cluster in the ventral part of the BNST was found to be significantly related with PIPS scores. Additionally, BNST volumes were also found to be related to clinical and third-wave cognitive measures which are known to be relevant in chronic pain and FM; more precisely (after applying multiple comparisons correction), highly significant associations between BNST volumes with functional impairment, depressive symptomatology, perceived stress, and the nonjudging mindfulness facet were observed. Regarding the longitudinal analyses, there was no differential effect of MBSR, compared to TAU, on BNST volumes. Participants showing higher increases in PIPS scores, indicative of a potentially negative impact on health, presented significant pre–post increases in BNST volumes, suggesting that changes in BNST volume across time may be related to changes in psychological inflexibility.

The BNST—part of “the extended amygdala”—is a key nucleus in the integration of autonomic and behavioral responses to stress; however, until recently it has been relatively overlooked compared to research examining amygdala functioning [48]. BNST integrates information from several upstream sources and, via dense connections with the paraventricular nucleus of the hypothalamus (PVN) (the principal node of the hypothalamic–pituitary–adrenal axis in initiating stress-mediated cortisol responses) regulates neuroendocrine and behavioral responses to stress [49]. The ventral part of the BNST has the largest density of noradrenergic fibers in the brain [50] and most of the projections to PVN originate from this area [51]. It has been proposed that the BNST extends the duration of fear response, enabling sustained vigilance (and so anxiety and perceived stress) characterized by temporally prolonged changes in arousal. This role contrasts with the amygdala, which regulates immediate fear responses to related to discrete and immediate threats [48]. The BNST has also been implicated in behavioral changes associated with depression; in particular, BNST activity has been related to anhedonic behavior after chronic mild stress, whereas lesions in this structure block the onset of learned helplessness in preclinical models [52].

Psychological inflexibility in pain, as measured by PIPS, comprises avoidance to pain [24], which is known to be inherently linked to fear of movement [23] and the anticipated threat value of intense pain. The ongoing relationship between fear of movement and pain avoidance results in perpetual vigilance and monitoring of sensations related to pain, promoting sensitization to low-intensity pain sensations, reducing mobility, and, ultimately, reduced function [53]. Regarding the implication of the BNST in the fear-avoidance model in chronic pain, a recent study by Meier et al. [54] reported that kinesiophobia is associated with brain activation (i.e., fMRI) in the BNST in patients with chronic low-back pain, compared to pain-free matched controls. In accord with these findings, our results support the notion of a role for BNST in conveying psychological inflexibility in chronic pain. Our finding of a positive association between BNST volume and self-reported perceived stress and depressive symptomatology, provides further support for a putative role for BNST in both stress response and allostasis [48,55]. 

We identified that BNST volumes positively correlated with the impact of FM, suggesting a potential role of this structure in the pathophysiology of the syndrome. BNST volumes also correlated with pain catastrophizing, a construct previously linked to pain intensity, depressive symptoms, disability, and delayed recovery in patients with chronic pain [56,57]. Pain catastrophizing can be conceptualized as a negative cognitive/affective response to anticipated or real pain; it comprises rumination upon, magnification of, and feelings of helplessness towards the pain experiences [57]. Pain catastrophizing has a crucial role in the fear avoidance model as it fosters fear-avoidance behaviors, which in turn result in exacerbation of physical and mental symptoms [56,58]. Alterations in GM morphology related to pain catastrophizing have been reported in patients with chronic pain, with associations found in brain areas involved in pain processing, emotion and motor activity, attention to pain, and top-down inhibition of pain, but not in BNST [59]. Our study suggests that BNST functioning may also contribute to the etiology of pain catastrophizing. Whether the role of BNST is unique to FM or more generally to other chronic painful disorders remains to be determined.

We also observed a significant negative association between BNST volumes and Nonjudging scores, suggesting that this structure may be involved in the neural basis of mindfulness. Given that mindfulness involves openness to the present experience (whether positively or negatively valent), a diminished role for brain areas implied in fear-anxiety-avoidance response might be expected in individuals with higher mindfulness scores. Mindfulness is a core component of psychological flexibility [15], accordingly, the association between Nonjudging scores and BNST volumes may also be due to a theoretical overlap between mindfulness and PIPS. The Nonjudging facet seems to be particularly related to the functional impact of FM [60] (more so than the other FFMQ subscales) and represents the component of acceptance in mindfulness as assessed by the FFMQ [38,61]. To the best of our knowledge, only one study has established before a relationship between this specific mindfulness facet and brain structure; more precisely, a positive association was found between Nonjudging and surface area in the superior prefrontal cortex in a non-clinical young sample [11], an area linked to self-referential processing, through introspection and self-awareness. In another study, dispositional mindfulness (assessed with the Mindfulness Attention and Awareness Scale) was negatively associated with amygdala volumes, a structure which—as we explained above—is closely related to the BNST [10].

Although reductions in BNST could be expected after the MBSR program, since this program promotes psychological flexibility [27], no effects were observed after the intervention. This null finding accords with previous studies looking for brain structural changes after MBSR; none reported changes in BNST [12]. Unexpectedly, the effect size of the intervention on PIPS scores was smaller than expected in the present sample, as significant changes with moderate-to-large effect sizes (d = 0.70) were found in the EUDAIMON study when including the whole study sample (*n* = 225) [27]. Here, PIPS scores had only a small-to-moderate effect size, (d = 0.42). It remains to be determined whether a more effective intervention (on PIPS scores) may have a greater impact on BNST volumes assessed post-intervention or at a later follow-up assessment. Interestingly, when we further explored changes in BNST volumes and their relation to changes in PIPS scores, we found that those patients experiencing higher increases in PIPS scores (i.e., increasing their psychological inflexibility), also showed increases in BNST volumes. This finding provides further support to the hypothesis that both PIPS and BNST are intrinsically related, perhaps even across time, and that an effective intervention that improves psychological flexibility may also affect BNST volumes.

Coming back to the lack of effect of MBSR on BNST volumes, it is worth bearing in mind that variations in GMV may not necessarily represent changes at the microstructure and cellular level [62], so we must not overlook that only part of the effects of the intervention on brain structure can be evaluated with the present design. Effect on other structural brain parameters (for example, white matter integrity), functional activity and connectivity of the BNST should also be evaluated in further better powered studies for comprehensively determine the effects of mindfulness on this specific brain cluster. Such studies also provide the desirable opportunity to examine structure–function relationships in BNST and their modulation by treatment.

Further research with larger samples is needed to confirm the role of the BNST in explaining PF and other key psychological constructs in patients with chronic pain conditions. Future studies should also ascertain whether the relationship between PF and BNST is exclusive of patients with FM or if this association is transdiagnostic. It is however surprising that the only brain cluster related to PIPS was found in the BNST and not in other brain areas frequently associated with PF-related constructs such as mindfulness facets or acceptance, for example, the anterior and posterior cingulate cortex/precuneus, insula, temporo–parietal junction, prefrontal cortex, hippocampus, and amygdala). Further studies with other clinical and non-clinical populations should also explore whether additional brain areas could also be related to PF. Likewise, case-control studies assessing whether clinical samples with greater psychological inflexibility (e.g. fibromyalgia) show indeed increased BNST volumes would be also welcome. Finally, longitudinal neuroimaging studies including interventions specifically targeting PF, such as Acceptance and Commitment Therapy, should also evaluate changes in BNST volumes to provide further evidence on the relevance of this area in conveying the effects of psychological treatments in chronic pain conditions.

Several limitations of the present study should be acknowledged. Firstly, the relatively small sample size limited our sensitivity to detect significant correlations, both at baseline and when seeking effects of treatment. Secondly, in our sample, MBSR did not produce a significant improvement in PIPS scores, reducing the likelihood of identifying corresponding changes in BNST volumes. Although the study focused in exploring neural correlates of PF in a sample of patients with FM, further studies should also include pain-free and/or clinical control participants to determine whether the relationship between BNST volumes and PF could be generalized to other chronic pain conditions or even to pain-free healthy individuals. 

## 5. Conclusions

It is well-known that psychological inflexibility plays a crucial role in mental health and chronic pain patients, and is an important mediator of the clinical effects of cognitive behavioral third-wave interventions. In a sample of patients with FM, we observed an association between psychological inflexibility to pain and GMV in a cluster in the ventral part of the BNST. This brain region correlated with clinical variables and third-wave cognitive process variables, suggesting a role for this area in both FM symptomatology and dispositional mindfulness. Although no effect of a mindfulness-based intervention on BNST was observed, increases in volume were found when considering participants reporting higher increases in PIPS scores. The findings of this study contribute to our understanding on the neurobiological bases of PF and its role in FM and encourage both the incorporation of PF as a core process measure in cross-sectional and clinical trials. Further, the novel identification of the importance of BNST in FM and potentially in other chronic painful disorders, may offer new directions to much-needed new therapies for pain. 

## Figures and Tables

**Table 1:** Sociodemographic and clinical characteristics of the study sample.

Variables	*N* = 47
Gender, *n* (female %)	47 (100%)
Age, M (SD)	51.60 (7.61)
Marital status, *n* (%)	
Single	2 (4.3%)
Married/living with a partner	36 (76.6%)
Separated/divorced	6 (12.8%)
Widowed	3 (6.4%)
Living arrangement, *n* (%)	
Living alone	3 (6.4%)
Living with partner	44 (93.6%)
Education level, *n* (%)	
Illiterate	2 (4.3%)
Did not graduate from primary school	1 (2.1%)
Primary school	22 (44.8%)
Secondary school	20 (42.6%)
University	2 (4.3%)
Employment status, *n* (%)	
Homemaker	3 (6.4%)
Paid employment	19 (40.4%)
Paid employment but in sick leave	1 (2.1%)
Unemployed with subsidy	4 (8.5%)
Unemployed without subsidy	10 (21.3%)
Retired/pensioner	6 (12.8%)
Temporal disability	0 (0.0%)
Others	4 (8.5%)
Clinical features	
Years of FM diagnosis, M (SD)	12.05 (8.76)
Current episode of depression, *n* (%)	27 (57.4%)
Previous episode(s) of depression, *n* (%)	20 (42.6%)
Dysthymia, *n* (%)	6 (12.8%)
Daily FM-related medication	
Analgesics, *n* (%)	10 (21.3%)
Anti-inflammatory, *n* (%)	7 (14.9%)
Opioids, *n* (%)	14 (29.8%)
Antiepileptic, *n* (%)	6 (12.8%)
Muscle relaxant, *n* (%)	1 (2.1%)
Antidepressants, *n* (%)	19 (40.4%)
Anxiolytics, *n* (%)	17 (36.2%)

Note: *n*= frequency, M= mean; SD= Standard deviation

**Table 2:** Means, standard deviations and correlations among main variables (i.e., PIPS and gray matter volumes in the BNST cluster, clinical and third-wave cognitive measures.

Main Variables	M (SD)	BNST Cluster Corr. (*p*)	BNST Theiss Corr. (*p*)	BNST Motzkin Corr. (*p*)
PIPS (12–84)	53.89 (16.51)	0.54 (<0.001)	*0.26 (0.089)*	*0.31 (0.036)*
FIQR (0–100)	60.99 (21.79)	0.47 (<0.001)	*0.32 (0.033)*	*0.35 (0.018)*
HADS-A (0–21)	10.21 (4.27)	*0.24 (0.106)*	0.14 (n.s.)	0.14 (n.s.)
HADS-D (0–21)	7.91 (5.21)	0.50 (<0.001)	*0.33 (0.026)*	*0.40 (0.007)*
PSS (0–40)	21.43 (10.64)	0.54 (<0.001)	*0.37 (0.012)*	*0.41 (0.005)*
PCS (0–52)	21.72 (13.45)	0.43 (0.003)	0.22 (n.s.)	*0.26 (0.088)*
FFMQ-Observing (8–40)	24.79 (6.22)	*0.35 (0.019)*	0.04 (n.s.)	0.08 (n.s.)
FFMQ-Describing (8–40)	27.17 (8.26)	*−0.26 (0.089)*	−0.19 (n.s.)	−0.22 (n.s.)
FFMQ-Acting with awareness (8–40)	26.09 (8.59)	*−0.30 (0.047)*	*−0.32 (0.032)*	*−0.33 (0.031)*
FFMQ-Non judging (8–40)	25.53 (8.62)	−0.49 (0.001)	*−0.33 (0.031)*	*−0.37 (0.015)*
FFMQ-Non reacting (7–35)	20.65 (5.95)	*−0.33 (0.028)*	*−0.27 (0.082)*	*−0.284 (0.061)*
SCS (1–5)	3.06 (0.99)	*−0.34 (0.026)*	*−0.24 (0.123)*	*−0.27 (0.074)*

Age and total GMV were entered as covariates in all correlations. Non-significant correlations after Bonferroni correction (i.e., *p* > 0.004) are shown in italics. Correlations between self-reported measures and BNST volumes using Theiss et al. [46] and Motzkin et al. [47] masks are also shown for comparative purposes. FFMQ = Five Facet Mindfulness Questionnaire; FIQR = Revised Fibromyalgia Impact Questionnaire; HADS = Hospital Anxiety and Depression Scale (A: Anxiety, D: Depression); BNST = Bed Nucleus of the Stria Terminalis; PCS = Pain Catastrophizing Scale; PIPS = Psychological Inflexibility in Pain Scale; PSS = Perceived Stress Scale; SCS = Self-Compassion Scale. n.s. = Not significant.

**Table 3:** Pre-post change in BNST volumes and PIPS scores following Mindfulness-Based Stress Reduction (MBSR) compared to Treatment-As-Usual (TAU) during 2-months.

	MBSR (*n* = 13)		TAU (*n* = 15)		Group × Time *p (ηp^2^)*
	PRE	POST	PRE	POST	
BNST	0.68 (0.05)	0.72 (0.05)	0.69 (0.05)	0.71 (0.07)	n.s. (0.03)
PIPS	57.69 (17.23)	52.77 (14.04)	51.53 (16.42)	50.53 (16.08)	n.s. (0.03)

Means and standard deviations (SD) are represented otherwise specified. BNST = Bed Nucleus of the Stria Terminalis; PIPS = Psychological Inflexibility in Pain Scale. n.s. = Not significant.
